# Accessibility and Content of Fellowship Programs for Cardiovascular Disease

**DOI:** 10.7759/cureus.25951

**Published:** 2022-06-15

**Authors:** Thai Donenfeld, Arjun Basnet, Chad Harris, Maham Waheed

**Affiliations:** 1 Internal Medicine, Maimonides Medical Center, New York City, USA

**Keywords:** website evaluation, accreditation council for graduate medical education (acgme), electronic residency application services (eras), fellowship content, content, cardiology interest, online presence, accessibility

## Abstract

Background

The objective of this study was to assess the accessibility and content of the Accreditation Council for Graduate Medical Education (ACGME)-accredited general cardiology fellowship websites.

Methods

Using the online information provided by the Electronic Residency Application Services (ERAS), we compiled a list of ACGME-accredited cardiac fellowship programs. The program links on ERAS were evaluated followed by a standard Google search of the program name + “cardiology fellowship”. Each program website was evaluated on the basis of program content, applying/recruiting and education.

Results

At the time of this study, we reviewed 231 general cardiology fellowship programs provided through ERAS. Of the 231 programs, 12 were excluded due to broken links, repeated links on ERAS, and websites with a general lack of content. We analyzed the data collected from the remaining 219 programs to assess the availability and general content of those websites. Data collected revealed a general lack of information regarding application processing and educational services but were sufficient in providing program descriptions and contact information.

Conclusions

ERAS can be used to locate general cardiology fellowships participating in the current match; however, the links provided by the program websites on ERAS are lacking in general content and accessibility. Although most websites did contain enough information about their program, there was a distinct lack of key information provided typically in the education services and application process.

## Introduction

General cardiology fellowship is mediated by the American College of Cardiology (ACC) and the American Heart Association (AHA). The ACC/AHA accreditation is “a single source of state-of-the-art process improvement tools to bridge gaps and integrate evidence-based science, quality initiatives, clinical best practices, and the latest ACC/AHA guidelines,” and serves as a guideline for cardiology fellowship program structure [1]. In 2015, the Accreditation Council for Graduate Medical Education (ACGME) set the minimum curricular standards and expectations for the approximately 190 accredited United States (US) fellowship programs (now over 200 programs) and still holds the standard for the core cardiology curriculum [2]. Access to proper information regarding the application process, clinical and research requirements, deadlines, program faculty, research prospects, and ease in fulfilling the required Core Cardiovascular Training Statement (COCATS) training is therefore essential for prospective fellows looking for a fellowship in general cardiology.

In our current climate of social distancing and virtual interviews, it is critical for fellowship programs to have working links, easy access, and adequate information on their web pages. A 2011 study by Chu [3] reported that 98% of anesthesiology applicants used anesthesia residency program websites (ARPW) but the ARPWs only had 46% of what was considered “useful”. That same study reported that 56% considering applying to their respective programs after visiting the websites [3]. Another 2005 study by Gaeta [4] showed that out of 222 emergency medicine residents, 78% reported that the residency website influenced their final decision. The same study also reported that at least 41% of applicants decided against a residency program based on that residency’s website [3]. The Gaeta study concluded that the residency website and its content were among the most important factors for influencing an applicant’s decision.

Website access and content have been previously studied for a variety of other fellowships, with a similar conclusion that the content of most programs does not provide an adequate amount of information for prospective applicants on their websites. A 2018 study by Shaath [5] demonstrated that the majority of orthopedic trauma surgery fellowships did not provide the reader enough information about the program. Another 2018 study by Cantrell [6] concluded that for abdominal transplant surgery fellowships a significant portion of fellowship websites did not provide functional links and of those that did, the websites contained inadequate information for potential candidates. A 2019 study by Ruddell [7] similarly concluded that for abdominal radiology fellowships, there was a discrepancy between the actual content the fellowship provided and what those programs presented online. Those studies concluded poor accessibility and content among most of the program websites. This is detrimental to applicant information and can lower the number of applicants for those programs.

The purpose of this study is to assess the content of ACGME-accredited general cardiology fellowship websites and the accessibility of that content.

## Materials and methods

The current list of general cardiology fellowships was taken from the Electronic Residency Application Services (ERAS) website [8]. The fellowships included were the general cardiology fellowships participating in 2019. Of the 233 programs available, 218 were participating at the time of the study. The sample was collected in two ways: first, an online search through a search engine (Google; Google LLC, Mountain View, California, United States) and, second, through the links provided on ERAS.

The program websites were then evaluated by a number of parameters, starting with the hyperlink, google location, program statement, program director, address, email and contact information, application information, and links to the ACC or AHA websites. The information obtained was then cross-referenced against the other 218 programs available: whether the links were present, present but not working, and not present. Once the hyperlinks were assessed, we also checked the program accessibility through Google. A specific Google search was performed for each program: [Program name as seen on ERAS] + “cardiology fellowship”, and the location of the link for the program was recorded as its location on Google. 

The content gathered from the program websites were evaluated for program overview, application process, and education. Program overview included the program description, the program director's name, address, email, phone number, current and previous fellows, and current employment of alumni. Application information included access to either the ACC or AHA with current guidelines, application process including the number of recommendations needed, deadlines, and salaries/benefits. Educational content included didactics, journal club, rotation/curriculum information, research requirements prior to entering the program, previous program research, any ongoing clinical trials occurring at the program, clinic/call responsibilities, case log requirements, and international opportunities. 

## Results

Of the total 231 general cardiology fellowships, we discounted 12 websites due to a total lack of any content, i.e., missing/no information at all, broken links, and inability to access the website or repeated links from ERAS. In total, we assessed the content and general accessibility of 219 general cardiology fellowships. Of the total amount of links provided, we were able to assess 94.8% for content of program overview, application process, and educational services. 

Google links provided by ERAS worked for 94.8% of the provided links. Using the standard search approach described above, we found general cardiology fellowship websites were the first available link on Google 83.5% of the time, with an average position for those websites on Google at 1.25.

Program information

Nearly all fellowship programs provided a program description (95.4%) and most of those programs also included a program director (92.6%). However, contact information and available access to the program was lacking. The phone number was widely given (96.3%); however, the hospital address was given by only 195 of the 219 (89%), and email information was only given by 204 (93.1%). When evaluating the data for current and past fellows, we found that programs provided their current fellows 68% of the time, and only 64 programs discussed past alumni (29.2%). Table 1 gives this data.

**Table 1:** Program information availability on fellowship program websites

Program Detail	Number of websites	Percentage of evaluated websites
Program description	209	95.4%
Program director	203	92.6%
Address	195	89.0%
Phone	211	96.3%
Email	204	93.1%
Current fellows	149	68.0%
Past fellows	64	29.2%

Application process

When evaluating the number of programs that provided links to the ACC or AHA websites, only 47 of the programs provided us with a link (21.4%). Of the 219, 195 provided information on the application process (89%) and 147 programs provided a deadline (67.1%). Fellowship salary was provided in 64 of the programs (29.2%). Table 2 gives this data.

**Table 2:** Application information available on general cardiology fellowship websites ACC: American College of Cardiology; AHA: American Heart Association

Program Detail	Number of websites	Percentage of evaluated websites
ACC/AHA Link	47	21.4%
Application process	195	89.0%
Deadline	147	67.1%
Salary	64	29.2%

Education services

Didactics were provided by 170 of the programs (77.6%) and a rotation schedule of some kind was provided by 139 programs (63.4%). Only 128 programs mentioned/provided links to current clinical trials or current program research (58.4%) and information regarding journal club was provided by 153 programs (69.8%). Information regarding research meetings and conferences was available in 139 programs (63.4%). Table 3 gives this data.

**Table 3:** Educational services available on fellowship websites

Program Detail	Number of Websites	Percent of evaluated websites
Didactics	170	77.6%
Journal club	153	69.8%
Rotation schedule	139	63.4%
Program research/clinical trials	128	58.4%
Research meeting/conferences	139	63.4%

## Discussion

Cardiovascular disease is currently the leading cause of mortality in the US, with the current estimation by the CDC being 610,000 deaths per year, roughly one in four deaths in the US [9]. According to a 2016 study by Nanrang et al., there is an increasing burden and growing demand for cardiovascular physicians. which must be optimized to meet the US public health needs [4]. Increasing the number of physicians treating cardiovascular disease is vital to addressing this demand. The Association for American Medical Colleges (AAMC) website has shown an increase in applications to cardiovascular disease fellowships from 2015 until 2018 [2]. The growing trend of interest in cardiovascular disease and previous data showing the influence of website content and application to those programs highlights an important dilemma in the decision making of prospective fellows and cardiology programs. This has become increasingly important during the 2020 coronavirus disease 2019 (COVID-19) pandemic. The importance of a strong online presence cannot be overstated as multiple programs have switched towards online interviews. Online meetings and virtual tours are becoming more prevalent, and due to the safety, convenience and saving costs from online interviews it has become key aspect in recruiting prospective fellows. 

Using ERAS, we evaluated 219 general cardiology fellowships after dismissing 12 websites for broken links and general lack of any content available (5.2%). Google search proved to be the best way to locate available programs (95.4%), with the average position of the program link at 1.24. Of the programs that were available to review for content, we learned that a majority of those programs included a program description (95.4%) and program director (92.6%). Contact information was generally available, as either a phone number or email was provided. However, even though the program director’s name was generally available, current and past fellows were not (68% and 29.2%, respectively). 

Similar studies in other specialties, including orthopedic trauma surgery, abdominal transplant surgery, and abdominal radiology fellowships, also demonstrated a lack of key information on their program websites. The number of applicants finding their information online is increasing, and this lack of content may prove deleterious to programs attempting to recruit the best applicants. When compared to our study on cardiology websites, this lack of information appears to trend through the studies previously described. A total of 17% of abdominal transplant surgery websites lacked contact details like phone numbers and emails [6] when compared to 4-7% of the cardiology websites. Cardiology websites also contained more information regarding cardiology didactic education (77.6%) when compared to abdominal transplant surgery didactic information (33%) [6]. However, unlike orthopedic trauma surgery [5], the cardiology websites did not reach 100% with descriptions and mission statements. The average of overview of rotation schedule was similar in both cardiology and orthopedic trauma surgery (63.5% and 65%, respectively) [5] and was significantly higher than abdominal transplant surgery, which had only 17% of their websites describe the fellow rotation schedule [6]. 

Program websites lacked general content and provided minimal information about their application process or educational services for prospective applicants. Application process was discussed by 89% of websites but only 21.4% of those websites provided a link to the ACC/AHA websites. Deadlines for applications were only provided by 67.1% of websites. Other data collected included educational services and research opportunities provided. Didactics and clinical schedules were provided in less than 80% of the programs listed. Much of the content regarding didactics and information on included board prep had minimal. Research meetings and conference was provided by 63.4% of programs and only 58.4% of programs included current research/clinical trials.

Our data does not show a total lack of general content provided by program websites, especially when compared to other fellowships reviewed in this paper. However, this study does highlight the discrepancies in information that exist. There is potential for access to better information in application processing and educational services.

Based on these analyses, we have developed a series of proposals to aid prospective fellows and fellowship program choose their desired applicant and program. First, websites should contain a description of the program followed by a picture and contact information of the program director. This will ensure that applicants know what the program is offering as well as the ability to contact the program director easily from the home page. Too often contact information is hidden behind multiple pages or links that limit ease of access for applicants with poor internet connections. Second, tabs/links about applications and requirements should be provided on one page. This decreases the amount of time applicants will be changing pages and increases ease of access to program content. Third, programs should inform applicants of the research opportunities that will be provided. This increases the likelihood of highly qualified applicants passionate about clinical research applying once they recognize a program's participation in research. Lastly, we do not recommend having a single page with all the program information as it increases website load time, increases clutter, and decreases accessibility to find specific information. A study by Portnet on e-commercial retail found that after the first one to two seconds, the conversion rate (buying a product) decreases by 0.3% per second and that "load speed for product category pages has the most impact on sales" [10]. For loading time of web pages, John Mueller, senior website trends analyst of Google, has recommended aiming for <2-3 secs [11]. While there is a clear distinction between shopping and fellowship application, the goal remains the same: how quickly can you spread information before the party loses interest.

## Conclusions

In conclusion, these data suggest general cardiology fellowships have a paucity of general content and accessibility provided online, which may pose a challenge to match applicants with desired programs. Multiple studies have shown that applicants find a majority of their fellowship information online and use that information to decide where to apply. Due to the ongoing pandemic it is vital that fellowship programs, including cardiology programs, have a strong online presence to recruit prospective fellows.
